# Supplementary material for: Carers’ and health workers’ perspectives on malnutrition in infants aged under six months in rural Ethiopia: A qualitative study
Source: PLoS One. 2022 Jul 21;17(7):e0271733. doi: 10.1371/journal.pone.0271733 (PMC9302717; doi:10.1371/journal.pone.0271733)
Supplement: S1 Text — (DOCX) [file pone.0271733.s001.docx]

**Annex I: Carer, community and health workers’ perspectives on malnutrition in infants aged under 6 months: a qualitative study**

**Interview Guide**

1. ***For mothers and community members***
2. What are the feeding practices for infants u6m in your family/area?

Probe with regard to:

- 1. Exclusive breast feeding
  2. Reasons for early initiation of complementary (supplementary) feeding (formula milk, animal milk, cereal based feeds, water, other food items, etc):
     1. Start early to avoid future problems
     2. Perceptions of Infant formula / other biscuits etc being “modern/progressive” thing to do
     3. General perceptions of formula (risks of formula)
  3. Use of animal milks
  4. Water
  5. Remedies for colic etc
  6. Giving foods which are available

1. Perceptions of “health / wellbeing” – what does an “ideal” infant look like?

Probe on the following issues

- 1. Feeding – how often, how long, how much (&How can you tell how much) should an infant feed?
  2. Sleep – how long and how often should an infant sleep?
  3. Behavior/activity (crying) – what should the ideal behavior or activity of an infant look like?
  4. Posseting (regurgitation) – how often should an infant regurgitate?
  5. Growth card / growth – when do you say an infant’s growth is normal?

1. What is your perception of health / wellbeing for the mothers & family?

Probe with regard to:

- 1. What is your perception on the nutrition / nutritious food for the mother
  2. How do you perceive SPENDING (or not spending money) on infant formula
  3. What is your perception on the thought that feeding breast milk may take time or may impact on mother’s work (i.e. stopping breast feeding to return to work)
  4. What is your thought on maternal well being (Stress / anxiety/sleep)

1. How does the community detect the manifestations of malnutrition

Probe with regard to:

- 1. What are the perceived reasons / causes of manifestations for malnutrition
  2. How is the care-seeking responses or characteristics looks like in this area

1. How is treatment for small infants aged below 6 months going on this area?

Probe:

- 1. What happens now?
  2. What would be “ideal” treatment?
  3. Preference for health facility – hospital? Health centre? Health post? Other?
  4. Social support systems – Who? How? Where? (including pregnancy)

1. Where is the source of information or sharing/availability
   1. Where they get their knowledge and understanding from
2. How could research project/other organizations working on nutrition for infants u6m best support infants/carers?
   1. KMC
   2. (Relaxation / stress reduction)
   3. (Pregnancy support)
3. How do you think COVID-19 could affect the health and well being of infants and mothers?

Probe with:

1. Transportation to health facilities, availability of routine services at health facilities (immunization, u5 visits, ANC/PNC, institutional deliveries, etc)

**I: Ilaalcha guddiftoonni, uummatnifi hojjettonni fayyaa hanqina nyaataa daa’imman ji’s 6 gadiirratti qaban: qo’annoo qaacessa haalaa**

**Qajeelcha Aafgaaffii**

1. ***Miseensota Uummataaf***
2. Hubannoofi beekumsa fayyaafi nageenya (“health / wellbeing”) – Daa’imni mudaa hin qabne (guutuu) tokko kan akkamiitii?

Qabxiilee armaan gadii soqi

- 1. Nyaacha – yeroo meeqaaf, yeroo hagam dheeratuuf, haga kam (fi hagam ta’uusaa akkamitti dubbatta) daa’imni tokko nyaachuu qaba?
  2. Rafiitii – yeroo hagamiif yeroo meeqa daa’imni tokko rafuu qabaaa?
  3. Amala/sochiilee (boo’icha) – amallii ykn sochiin daa’ima mudaa hinqabnee tokko maal ta’uu qaba?
  4. Deeffachuu – daa’imni kichuun tokko yeroo meeqa deeffachuu qabdi?
  5. Kaardii guddinaa/guddina – yoo maal ta’e guddinni daa’ima tokkoo haala gaarii irra jira jettu?

1. Hubannoofi beekumsa keessan waa’ee fayyaafi nageenya haadholiifi maatii

Kanneen soqamuu qaban:

- 1. Hubannoofi beekumsi kee waa’ee nyaata haadhoolii irratti qabdu maali?
  2. Nyaata daa’immani warshaan qophaa’anii dhiyaatanii (foormulaa)f maallaqa baasuufi baasuu dhiisuu akkamitti ilaalta?
  3. Yaada daa’ima harma haadhaa hoosisuun yeroo fixa ykn haadha huba jedhu akkamitti ilaalta? (jechuun hojii deeb’uuf hoosisuu dhaabuu/harma guuss)
  4. Nagaaf gammachuu (wellbeing) haadhoolii irratti yaada akkamii qabda? (Dhiphina/cinqii/rafiitii)

1. Maatii ykn naannoo keessanitti gochaalee nyaachisa daa’imman ji’a 6 gadii maalfaadha?

Kanneen soqamuu qaban:

- 1. Harma haadhaa qofa hoosisuu
  2. Sababiin nyaata warshaan qophaa’an/foormulaa (nyaata dabalataa):
     1. Rakkoolee fulduratti mudachuu danda’an qolachuuf
     2. Nyaata warshaan qophaa’an (foormulaa daa’immanii) kanneen akka buskutaa nyaachisuu akka ammayyummaa/qaroominaatti ilaaluu
     3. Beekumsaafi ilaacha waliigalaa nyaata waarshaan qopheeffamanii/foormulaa (gaaga’umsa foormulaa)
  3. Aannan beelladaa fayyadamuu
  4. Bishaan
  5. Furmaata dhibee garaaf kkf
  6. Nyaata argame kennuufii

1. Uummatni mul’achuu hanqina nyaataa akkamitti adda baafataa?

Kanneen soqi:

- 1. Akka illacha isaaniitti sababiiwwan/ka’umsi mul’achuu hanqina nyaataa maalfaati?
  2. Akkaataan ykn ammalli kunuunsa/yaalii barbaaddachuu naannoo kanaa maal fakkaata?

1. Wal’aansi/yaaliin daa’ima xiqqoo ji’a 6 gadii naannoo kanatti akkamiin deemaa jira?

Soqi:

- 1. Yeroo ammaa kana maaltu ta’aa jira?
  2. Wal’aanis/yaaliin mudaa hin qabne (guutuun) maal ta’uu qaba?
  3. Filannoo wiirtuu tajaajila fayyaa– hospitala? Buufata fayyaa? Keellaa fayyaa? Kannne biroo?
  4. Sirna deeggarsa hawaasumma – Eenyu? Akkamitti? Eessatti? (ulfa dabalatee)

1. Maddeen odeeffannoo itti argamu ykn irraa qooddatan eessaatti?
   1. Beekumsaafi hubannoo eessaaa argatuu?
2. Qo’annoon haala nyaata daa’imman ji’s 6 gadiirratti taasifamu akkamitti daa’imman ykn guddistoota deeggaruu danda’aa?
   1. Kunuunsa haadha kaangaaroo (KMC)
   2. (Bashannana / dhipphina hir’isuu)
   3. (Deeggarsa ulfaa)
3. Kooviid-19 akkamitti fayyaafi nageenya daa’imman kichuufi haadholii miidhuu danda’a jettee yaadda?

Soqi:

Gara bu’uuraalee fayyaa imaluufi tajaajila argachuu irratti (talaallii wagaa 5 gadi, HDD/HDB, dhaabbilee fayyaatti da’uu, kkf)
