# Supplementary material for: Carers’ and health workers’ perspectives on malnutrition in infants aged under six months in rural Ethiopia: A qualitative study
Source: PLoS One. 2022 Jul 21;17(7):e0271733. doi: 10.1371/journal.pone.0271733 (PMC9302717; doi:10.1371/journal.pone.0271733)
Supplement: S2 Text — (DOCX) [file pone.0271733.s002.docx]

**Annex II: Carer, community and health workers’ perspectives on malnutrition in infants aged under 6 months: a qualitative study**

***For health care workers (HCWs)***

1. What are the feeding practices for infants under 6months in this area

Probe with regard to:

- 1. Exclusive breast feeding
     1. Reasons for early initiation of complementary (supplementary) feeding (formula milk, animal milk, cereal based feeds, water, other food items):
     2. Start early to avoid future problems
     3. Perceptions of Infant formula / other biscuits etc being “modern/progressive” thing to do
     4. General perceptions of formula (risks of formula)
  2. Use of animal milks
  3. Water
  4. Remedies for colic etc
  5. Giving foods which are available

1. What is your perceptions of “health / wellbeing” – what does an “ideal” infant look like?

Probe with regard to:

- 1. Feeding – how often, how long, how much should an infant feed?
  2. Sleep – how long and how often should an infant sleep?
  3. Behavior/activity (crying) – what should the ideal behavior or activity of an infant look like?
  4. Posseting (regurgitation) – how often should an infant regurgitate?
  5. Growth card / growth – when do you say an infant’s growth is normal?

1. What is your perceptions of health / wellbeing for mother & family

Probe with regard to:

- 1. What is your perception on the Nutrition / nutritious food for the mother
  2. How do you see SPENDING (or not spending money) on infant formula
  3. What is your perception on the thought that feeding breast milk may take time or may Impact on mother’s work (i.e. stopping breast feeding to return to work)
  4. How do you understand maternal well being (Stress / anxiety/sleep)

1. How do you make detection for manifestations of malnutrition (“when and how can you say if an infant u6m is malnourished”)

Probing questions:

- 1. Perceived reasons / causes
  2. Weight and weight-for-length measurement (how easy, how quick, how commonly done; how often missed; problems with scales & equipment)
  3. MUAC for u6 months – experience with MUAC for older children? How easy might this be for infants <6m? How quick? Which measure would they prefer?
  4. Other symptoms?

1. What are the treatment option for small infants under 6months
   1. What happens now– what do you do now if you find a small baby?
   2. What are current challenges to managing small infants / those with feeding problems
      1. Do carers accept referral into hospital (as per current guidelines) – any experience?
      2. Time taken to advise & counsel
   3. What do you think are possible solutions? / What would be “ideal” treatment
      1. Where should treatment mainly be done?
      2. Who should be main contact point? (would nurses be OK?)
      3. What training / support would be needed to help you / colleagues better look after small infants <6m
   4. Social support systems – who? How? Where? (including pregnancy)
      1. What support groups / organization etc exist which we could link to?
      2. What happens with infants who cannot BF?
2. What are the sources of information sharing/availability
   1. Where do HCW get their knowledge and understanding from
      1. In initial training?
      2. In follow-up / on-the-job training?
   2. Skills gaps – are HCW adequately trained? What training would be desirable
3. How can research project designed on nutrition among infant u6m best support infants / carers?
   1. *(CMAMI tool)*
   2. KMC
   3. (Relaxation / stress reduction)
   4. (Pregnancy support)
4. How do you think COVID-19 could affect the health and well being of infants and mothers as well as such research projects? Probe with:
5. Transportation to health facilities, availability of routine services at health facilities (immunization, u5 visits, ANC/PNC, institutional deliveries, etc)
6. ***Hojjettota Kunuunsa Fayyaatiif (HCWs)***
7. Hubannoofi beekumsa fayyaafi nageenya (“health / wellbeing”) – Daa’imni mudaa hin qabne (guutuu) tokko kan akkamiitii?

Qabxiilee armaan gadii soqi

- 1. Nyaacha – yeroo meeqaaf, yeroo hagam dheeratuuf, haga kam (fi hagam ta’uusaa akkamitti dubbatta) daa’imni tokko nyaachuu qaba?
  2. Rafiitii – yeroo hagamiif yeroo meeqa daa’imni tokko rafuu qabaaa?
  3. Amala/sochiilee (boo’icha) – amallii ykn sochiin daa’ima mudaa hinqabnee tokko maal ta’uu qaba?
  4. Deeffachuu – daa’imni kichuun tokko yeroo meeqa deeffachuu qabdi?
  5. Kaardii guddinaa/guddina – yoo maal ta’e guddinni daa’ima tokkoo haala gaarii irra jira jettu?

1. Hubannoofi beekumsa keessan waa’ee fayyaafi nageenya haadholiifi maatii

Kanneen soqamuu qaban:

- 1. Hubannoofi beekumsi kee waa’ee nyaata haadhoolii irratti qabdu maali?
  2. Nyaata daa’immani warshaan qophaa’anii dhiyaatanii (foormulaa)f maallaqa baasuufi baasuu dhiisuu akkamitti ilaalta?
  3. Yaada daa’ima harma haadhaa hoosisuun yeroo fixa ykn haadha huba jedhu akkamitti ilaalta? (jechuun hojii deeb’uuf hoosisuu dhaabuu/harma guuss)
  4. Nagaaf gammachuu (wellbeing) haadhoolii irratti yaada akkamii qabda? (Dhiphina/cinqii/rafiitii)

1. Maatii ykn naannoo keessanitti gochaalee nyaachisa daa’imman ji’a 6 gadii maalfaadha?

Kanneen soqamuu qaban:

- 1. Harma haadhaa qofa hoosisuu
  2. Sababiin nyaata warshaan qophaa’an/foormulaa (nyaata dabalataa):
     1. Rakkoolee fulduratti mudachuu danda’an qolachuuf
     2. Nyaata warshaan qophaa’an (foormulaa daa’immanii) kanneen akka buskutaa nyaachisuu akka ammayyummaa/qaroominaatti ilaaluu
     3. Beekumsaafi ilaacha waliigalaa nyaata waarshaan qopheeffamanii/foormulaa (gaaga’umsa foormulaa)
  3. Aannan beelladaa fayyadamuu
  4. Bishaan
  5. Furmaata dhibee garaaf kkf
  6. Nyaata argame kennuufii

1. Mul’achuu hanqina nyaataa adda baafachuuf maal goota (“yoomiifi akamitti daa’imni ji’a 6 gadii tokko hanqina nyaataa qaba jetta”)

Gaaffilee soqaatii:

- 1. Sababa/ka’umsa akka hubannoo ufiitti
  2. Ulfaatinaafi ulfaatina hojjaaf safaruu/Weight and weight-for-length measurement (akkataa salphina, saffisa, irra deddebiin hojjechuu, yeroo meeqa dhabama, rakkoolee safartuuwwaniifi meeshaalee)
  3. Maraa walakkaa ciqilee olii (MUAC) daa’imman ji’a 6 gadiif – muuxannoo MUAC daa’imman jajjaboof? Daa’imman ji’a 6 gaaddiif hagam salphachuu danda’a? Saffisa akkamiin? Safartuu isa kaam filatu laata?
  4. Mallattoolee biroo?

1. Daa’iimman xixiqqoo ji’s 6 gadiif filannoo wal’aansaa/yaalii maaltu jira?
   1. Yeroo ammaa kana maaltu ta’aa jira– amma osoo daa’ima xiqqoo argitee maal goota?
   2. Yeroo ammaa kanatti daa’imman xixxiqqoo rakkoolee nyaataa qaban wal’aanuu keessatti rakkooleen jiran maalfadhi?
      1. Kunuunstonni/guddistoonni riiferii gara hospitaalaatti kennamuuf nifudhtu (akkataa qajeelfama amma jiruutiin) – muuxannoo wayii yoo jiraate?
      2. Gorsuufi qajeelchuuf yeroo inni fudhatu.
   3. Furmaatileen jirhu jettee yaaddaa? Wal’aansi mudaa hin qabne/guutuu ta’e maal laata?
      1. Wal’ansi eessatti godhamuu qaba?
      2. Bakki walqunnaman eenyu ta’uu qaba (Narsoonni ni ta’uu lataa?)
      3. Daa’imman ji’a 6 gadi bifa fooyya’an akka taasiftaniin leenjii/deeggarsa akkamiitu isin barbaachisa?
   4. Sirna deeggarsa hawwaasummaa – Eenyu? Akkamitti? Eessatti? (ulfa dabalatee)
      1. Garee/dhaabbilee deegarsaa nuti qunnamu qabnu maaliitu jira?
      2. Daa’ima harma haadhaa hodhuu hin dandeenye maaltu mudata?
2. Maddeen odeeffannoo itti argamu ykn irraa qooddatan eessaatti?
   1. HEF beekumsaafi hubannaa eessaa argatu?
      1. Leenjii isa jalqabaatii?
      2. Leenjii hordoffii/hojiirraatii?
   2. Qaawa/hir’ina dandeettiiwwanii – HEF leenjii ga’aa fudhataniiruu? Leenjii akkamiitu barbaachisaa?

7) Qo’annoon haala nyaata daa’imman ji’s 6 gadiirratti taasifamu akkamitti daa’imman ykn guddistoota deeggaruu danda’aa?

- 1. Meeshaa cMAMI *(CMAMI tool)*
  2. Kunuunsa haadha kaangaaroo (KMC)
  3. (Bashannana / dhipphina hir’isuu)
  4. (Deeggarsa ulfaa)

1. Kooviid-19 akkamitti fayyaafi nageenya daa’imman kichuufi haadholii miidhuu danda’a jettee yaadda?

Soqi:

Gara bu’uuraalee fayyaa imaluufi tajaajila argachuu irratti (talaallii wagaa 5 gadi, HDD/HDB, dhaabbilee fayyaatti da’uu, kkf)
