# Supplementary material for: Carers’ and health workers’ perspectives on malnutrition in infants aged under six months in rural Ethiopia: A qualitative study
Source: PLoS One. 2022 Jul 21;17(7):e0271733. doi: 10.1371/journal.pone.0271733 (PMC9302717; doi:10.1371/journal.pone.0271733)
Supplement: S1 File — (DOCX) [file pone.0271733.s003.docx]

**I: Ilaalcha guddiftoonni, uummatnifi hojjettonni fayyaa hanqina nyaataa daa’imman ji’s 6 gadiirratti qaban: qo’annoo qaacessa haalaa**

**Qajeelcha Aafgaaffii**

1. ***Miseensota Uummataaf***
2. Hubannoofi beekumsa fayyaafi nageenya (“health / wellbeing”) – Daa’imni mudaa hin qabne (guutuu) tokko kan akkamiitii?

Qabxiilee armaan gadii soqi

- 1. Nyaacha – yeroo meeqaaf, yeroo hagam dheeratuuf, haga kam (fi hagam ta’uusaa akkamitti dubbatta) daa’imni tokko nyaachuu qaba?
  2. Rafiitii – yeroo hagamiif yeroo meeqa daa’imni tokko rafuu qabaaa?
  3. Amala/sochiilee (boo’icha) – amallii ykn sochiin daa’ima mudaa hinqabnee tokko maal ta’uu qaba?
  4. Deeffachuu – daa’imni kichuun tokko yeroo meeqa deeffachuu qabdi?
  5. Kaardii guddinaa/guddina – yoo maal ta’e guddinni daa’ima tokkoo haala gaarii irra jira jettu?

1. Hubannoofi beekumsa keessan waa’ee fayyaafi nageenya haadholiifi maatii

Kanneen soqamuu qaban:

- 1. Hubannoofi beekumsi kee waa’ee nyaata haadhoolii irratti qabdu maali?
  2. Nyaata daa’immani warshaan qophaa’anii dhiyaatanii (foormulaa)f maallaqa baasuufi baasuu dhiisuu akkamitti ilaalta?
  3. Yaada daa’ima harma haadhaa hoosisuun yeroo fixa ykn haadha huba jedhu akkamitti ilaalta? (jechuun hojii deeb’uuf hoosisuu dhaabuu/harma guuss)
  4. Nagaaf gammachuu (wellbeing) haadhoolii irratti yaada akkamii qabda? (Dhiphina/cinqii/rafiitii)

1. Maatii ykn naannoo keessanitti gochaalee nyaachisa daa’imman ji’a 6 gadii maalfaadha?

Kanneen soqamuu qaban:

- 1. Harma haadhaa qofa hoosisuu
  2. Sababiin nyaata warshaan qophaa’an/foormulaa (nyaata dabalataa):
     1. Rakkoolee fulduratti mudachuu danda’an qolachuuf
     2. Nyaata warshaan qophaa’an (foormulaa daa’immanii) kanneen akka buskutaa nyaachisuu akka ammayyummaa/qaroominaatti ilaaluu
     3. Beekumsaafi ilaacha waliigalaa nyaata waarshaan qopheeffamanii/foormulaa (gaaga’umsa foormulaa)
  3. Aannan beelladaa fayyadamuu
  4. Bishaan
  5. Furmaata dhibee garaaf kkf
  6. Nyaata argame kennuufii

1. Uummatni mul’achuu hanqina nyaataa akkamitti adda baafataa?

Kanneen soqi:

- 1. Akka illacha isaaniitti sababiiwwan/ka’umsi mul’achuu hanqina nyaataa maalfaati?
  2. Akkaataan ykn ammalli kunuunsa/yaalii barbaaddachuu naannoo kanaa maal fakkaata?

1. Wal’aansi/yaaliin daa’ima xiqqoo ji’a 6 gadii naannoo kanatti akkamiin deemaa jira?

Soqi:

- 1. Yeroo ammaa kana maaltu ta’aa jira?
  2. Wal’aanis/yaaliin mudaa hin qabne (guutuun) maal ta’uu qaba?
  3. Filannoo wiirtuu tajaajila fayyaa– hospitala? Buufata fayyaa? Keellaa fayyaa? Kannne biroo?
  4. Sirna deeggarsa hawaasumma – Eenyu? Akkamitti? Eessatti? (ulfa dabalatee)

1. Maddeen odeeffannoo itti argamu ykn irraa qooddatan eessaatti?
   1. Beekumsaafi hubannoo eessaaa argatuu?
2. Qo’annoon haala nyaata daa’imman ji’s 6 gadiirratti taasifamu akkamitti daa’imman ykn guddistoota deeggaruu danda’aa?
   1. Kunuunsa haadha kaangaaroo (KMC)
   2. (Bashannana / dhipphina hir’isuu)
   3. (Deeggarsa ulfaa)
3. Kooviid-19 akkamitti fayyaafi nageenya daa’imman kichuufi haadholii miidhuu danda’a jettee yaadda?

Soqi:

Gara bu’uuraalee fayyaa imaluufi tajaajila argachuu irratti (talaallii wagaa 5 gadi, HDD/HDB, dhaabbilee fayyaatti da’uu, kkf)
