# Supplementary material for: Carers’ and health workers’ perspectives on malnutrition in infants aged under six months in rural Ethiopia: A qualitative study
Source: PLoS One. 2022 Jul 21;17(7):e0271733. doi: 10.1371/journal.pone.0271733 (PMC9302717; doi:10.1371/journal.pone.0271733)
Supplement: S2 File — (DOCX) [file pone.0271733.s004.docx]

1. ***Hojjettota Kunuunsa Fayyaatiif (HCWs)***
2. Hubannoofi beekumsa fayyaafi nageenya (“health / wellbeing”) – Daa’imni mudaa hin qabne (guutuu) tokko kan akkamiitii?

Qabxiilee armaan gadii soqi

- 1. Nyaacha – yeroo meeqaaf, yeroo hagam dheeratuuf, haga kam (fi hagam ta’uusaa akkamitti dubbatta) daa’imni tokko nyaachuu qaba?
  2. Rafiitii – yeroo hagamiif yeroo meeqa daa’imni tokko rafuu qabaaa?
  3. Amala/sochiilee (boo’icha) – amallii ykn sochiin daa’ima mudaa hinqabnee tokko maal ta’uu qaba?
  4. Deeffachuu – daa’imni kichuun tokko yeroo meeqa deeffachuu qabdi?
  5. Kaardii guddinaa/guddina – yoo maal ta’e guddinni daa’ima tokkoo haala gaarii irra jira jettu?

1. Hubannoofi beekumsa keessan waa’ee fayyaafi nageenya haadholiifi maatii

Kanneen soqamuu qaban:

- 1. Hubannoofi beekumsi kee waa’ee nyaata haadhoolii irratti qabdu maali?
  2. Nyaata daa’immani warshaan qophaa’anii dhiyaatanii (foormulaa)f maallaqa baasuufi baasuu dhiisuu akkamitti ilaalta?
  3. Yaada daa’ima harma haadhaa hoosisuun yeroo fixa ykn haadha huba jedhu akkamitti ilaalta? (jechuun hojii deeb’uuf hoosisuu dhaabuu/harma guuss)
  4. Nagaaf gammachuu (wellbeing) haadhoolii irratti yaada akkamii qabda? (Dhiphina/cinqii/rafiitii)

1. Maatii ykn naannoo keessanitti gochaalee nyaachisa daa’imman ji’a 6 gadii maalfaadha?

Kanneen soqamuu qaban:

- 1. Harma haadhaa qofa hoosisuu
  2. Sababiin nyaata warshaan qophaa’an/foormulaa (nyaata dabalataa):
     1. Rakkoolee fulduratti mudachuu danda’an qolachuuf
     2. Nyaata warshaan qophaa’an (foormulaa daa’immanii) kanneen akka buskutaa nyaachisuu akka ammayyummaa/qaroominaatti ilaaluu
     3. Beekumsaafi ilaacha waliigalaa nyaata waarshaan qopheeffamanii/foormulaa (gaaga’umsa foormulaa)
  3. Aannan beelladaa fayyadamuu
  4. Bishaan
  5. Furmaata dhibee garaaf kkf
  6. Nyaata argame kennuufii

1. Mul’achuu hanqina nyaataa adda baafachuuf maal goota (“yoomiifi akamitti daa’imni ji’a 6 gadii tokko hanqina nyaataa qaba jetta”)

Gaaffilee soqaatii:

- 1. Sababa/ka’umsa akka hubannoo ufiitti
  2. Ulfaatinaafi ulfaatina hojjaaf safaruu/Weight and weight-for-length measurement (akkataa salphina, saffisa, irra deddebiin hojjechuu, yeroo meeqa dhabama, rakkoolee safartuuwwaniifi meeshaalee)
  3. Maraa walakkaa ciqilee olii (MUAC) daa’imman ji’a 6 gadiif – muuxannoo MUAC daa’imman jajjaboof? Daa’imman ji’a 6 gaaddiif hagam salphachuu danda’a? Saffisa akkamiin? Safartuu isa kaam filatu laata?
  4. Mallattoolee biroo?

1. Daa’iimman xixiqqoo ji’s 6 gadiif filannoo wal’aansaa/yaalii maaltu jira?
   1. Yeroo ammaa kana maaltu ta’aa jira– amma osoo daa’ima xiqqoo argitee maal goota?
   2. Yeroo ammaa kanatti daa’imman xixxiqqoo rakkoolee nyaataa qaban wal’aanuu keessatti rakkooleen jiran maalfadhi?
      1. Kunuunstonni/guddistoonni riiferii gara hospitaalaatti kennamuuf nifudhtu (akkataa qajeelfama amma jiruutiin) – muuxannoo wayii yoo jiraate?
      2. Gorsuufi qajeelchuuf yeroo inni fudhatu.
   3. Furmaatileen jirhu jettee yaaddaa? Wal’aansi mudaa hin qabne/guutuu ta’e maal laata?
      1. Wal’ansi eessatti godhamuu qaba?
      2. Bakki walqunnaman eenyu ta’uu qaba (Narsoonni ni ta’uu lataa?)
      3. Daa’imman ji’a 6 gadi bifa fooyya’an akka taasiftaniin leenjii/deeggarsa akkamiitu isin barbaachisa?
   4. Sirna deeggarsa hawwaasummaa – Eenyu? Akkamitti? Eessatti? (ulfa dabalatee)
      1. Garee/dhaabbilee deegarsaa nuti qunnamu qabnu maaliitu jira?
      2. Daa’ima harma haadhaa hodhuu hin dandeenye maaltu mudata?
2. Maddeen odeeffannoo itti argamu ykn irraa qooddatan eessaatti?
   1. HEF beekumsaafi hubannaa eessaa argatu?
      1. Leenjii isa jalqabaatii?
      2. Leenjii hordoffii/hojiirraatii?
   2. Qaawa/hir’ina dandeettiiwwanii – HEF leenjii ga’aa fudhataniiruu? Leenjii akkamiitu barbaachisaa?

7) Qo’annoon haala nyaata daa’imman ji’s 6 gadiirratti taasifamu akkamitti daa’imman ykn guddistoota deeggaruu danda’aa?

- 1. Meeshaa cMAMI *(CMAMI tool)*
  2. Kunuunsa haadha kaangaaroo (KMC)
  3. (Bashannana / dhipphina hir’isuu)
  4. (Deeggarsa ulfaa)

1. Kooviid-19 akkamitti fayyaafi nageenya daa’imman kichuufi haadholii miidhuu danda’a jettee yaadda?

Soqi:

Gara bu’uuraalee fayyaa imaluufi tajaajila argachuu irratti (talaallii wagaa 5 gadi, HDD/HDB, dhaabbilee fayyaatti da’uu, kkf)
