# Supplementary material for: Carers’ and health workers’ perspectives on malnutrition in infants aged under six months in rural Ethiopia: A qualitative study
Source: PLoS One. 2022 Jul 21;17(7):e0271733. doi: 10.1371/journal.pone.0271733 (PMC9302717; doi:10.1371/journal.pone.0271733)
Supplement: Attachment — Submitted filename: Response to Reviewers.doc [file pone.0271733.s005.doc]

**Point by point response to reviewers’ and editors’ comments, questions and suggestions**

Manuscript ID: PONE-D-21-13630

**Carers’ and health workers’ perspectives on malnutrition in infants aged under six months in rural Ethiopia: A qualitative study**

Thanking the reviewers and editors for their invaluable comments and suggestions, we hereby provide point by point responses to the comments, suggestions and questions.

Journal Requirements:

When submitting your revision, we need you to address these additional requirements.

1. Please ensure that your manuscript meets PLOS ONE's style requirements, including those for file naming. The PLOS ONE style templates can be found at
   <https://journals.plos.org/plosone/s/file?id=wjVg/PLOSOne_formatting_sample_main_body.pdf> and 
   <https://journals.plos.org/plosone/s/file?id=ba62/PLOSOne_formatting_sample_title_authors_affiliations.pdf>

**Response:** We confirm our manuscript meets PLOS ONE’s style requirements.

2. Please include a copy of the interview guide used in the study, in both the original language and English, as Supporting Information, or include a citation if it has been published previously.

**Response:** Included

3. In your Data Availability statement, you have not specified where the minimal data set underlying the results described in your manuscript can be found. PLOS defines a study's minimal data set as the underlying data used to reach the conclusions drawn in the manuscript and any additional data required to replicate the reported study findings in their entirety. All PLOS journals require that the minimal data set be made fully available. For more information about our data policy, please see <http://journals.plos.org/plosone/s/data-availability>.

Upon re-submitting your revised manuscript, please upload your study’s minimal underlying data set as either Supporting Information files or to a stable, public repository and include the relevant URLs, DOIs, or accession numbers within your revised cover letter. For a list of acceptable repositories, please see [http://journals.plos.org/plosone/s/data-availability#loc-recommended-repositories](http://journals.plos.org/plosone/s/data-availability#loc-recommended-repositories). Any potentially identifying patient information must be fully anonymized.

Important: If there are ethical or legal restrictions to sharing your data publicly, please explain these restrictions in detail. Please see our guidelines for more information on what we consider unacceptable restrictions to publicly sharing data: [http://journals.plos.org/plosone/s/data-availability#loc-unacceptable-data-access-restrictions](http://journals.plos.org/plosone/s/data-availability#loc-unacceptable-data-access-restrictions). Note that it is not acceptable for the authors to be the sole named individuals responsible for ensuring data access.

We will update your Data Availability statement to reflect the information you provide in your cover letter.

**Response:** We could not upload the dataset or put it on a public repository since we didn’t obtain the permission to do so during the data collection (both from the participants and the ethics committees). However, the dataset can be obtained from the corresponding author on reasonable requests.

4. PLOS requires an ORCID iD for the corresponding author in Editorial Manager on papers submitted after December 6th, 2016. Please ensure that you have an ORCID iD and that it is validated in Editorial Manager. To do this, go to ‘Update my Information’ (in the upper left-hand corner of the main menu), and click on the Fetch/Validate link next to the ORCID field. This will take you to the ORCID site and allow you to create a new iD or authenticate a pre-existing iD in Editorial Manager. Please see the following video for instructions on linking an ORCID iD to your Editorial Manager account: <https://www.youtube.com/watch?v=_xcclfuvtxQ>

**Response:** Corresponding author’s ORCID iD was already mentioned during first submission.

5. Your abstract cannot contain citations. Please only include citations in the body text of the manuscript and ensure that they remain in ascending numerical order on first mention.

**Response:** We confirm our abstract does not contain any citations.

**Reviewers' comments:**

Reviewer's Responses to Questions

**Comments to the Author**

1. Is the manuscript technically sound, and do the data support the conclusions?

The manuscript must describe a technically sound piece of scientific research with data that supports the conclusions. Experiments must have been conducted rigorously, with appropriate controls, replication, and sample sizes. The conclusions must be drawn appropriately based on the data presented.

Reviewer #1: Yes

Reviewer #2: Yes

Reviewer #3: Partly

**Response:** Thanks, we have edited the conclusion part

2. Has the statistical analysis been performed appropriately and rigorously?

Reviewer #1: N/A

Reviewer #2: N/A

Reviewer #3: N/A

3. Have the authors made all data underlying the findings in their manuscript fully available?

The [PLOS Data policy](http://www.plosone.org/static/policies.action#sharing) requires authors to make all data underlying the findings described in their manuscript fully available without restriction, with rare exception (please refer to the Data Availability Statement in the manuscript PDF file). The data should be provided as part of the manuscript or its supporting information, or deposited to a public repository. For example, in addition to summary statistics, the data points behind means, medians and variance measures should be available. If there are restrictions on publicly sharing data—e.g. participant privacy or use of data from a third party—those must be specified.

Reviewer #1: Yes

Reviewer #2: Yes

Reviewer #3: No

**Response:** We could not upload the dataset or put it on a public repository since we did not obtain the permission to do so during the data collection (from both, the participants and the ethics committees). However, the dataset can be obtained from the corresponding author on reasonable requests.

4. Is the manuscript presented in an intelligible fashion and written in standard English?

PLOS ONE does not copyedit accepted manuscripts, so the language in submitted articles must be clear, correct, and unambiguous. Any typographical or grammatical errors should be corrected at revision, so please note any specific errors here.

Reviewer #1: Yes

Reviewer #2: Yes

Reviewer #3: Yes

5. Review Comments to the Author

Please use the space provided to explain your answers to the questions above. You may also include additional comments for the author, including concerns about dual publication, research ethics, or publication ethics. (Please upload your review as an attachment if it exceeds 20,000 characters)

**Reviewer #1:**

1. General comments: Good study to assess understanding in the community perspective especially in preparation for a trial. The results section can be shortened by combining HW and Community members perspectives. As it is now there is a lot of repetition that can be avoided. The authors can consider presenting the common responses from the two groups whilst still highlighting differences in opinion between the two groups. The results section is currently 18 pages and difficult to get through. This can be shortened to improve on its readership.

**Response:** We agree the results section appears longer and we acknowledge the reviewer’s suggestion of merging some of the findings. We have merged some of the findings from the different categories of participants. However, we did not do so throughout since merging the views of the different categories of the participants might lead to distortion of the flow and organization of the manuscript and might somehow mislead readers.

2. In the methods section, I take note of the fact that the data was collected by 3 people. The authors should consider clarifying why this was necessary especially because it implicates reliability and validity of the data. Clarify how did you decide on who interviews who?

**Response:** All interviews were conducted by a team of individuals, a sociologist who facilitated all interviews, supported by a pediatrician and a child nutrition expert. The pediatrician did not conduct interviews of caretaker/parents of sick children and health workers working in Jimma Medical Center where he works to avoid influence of the patient-physician power relationship on the interview process and data quality. The child nutrition expert was involved in interviews with Amharic speaking participants only since he does not command Afaan Oromo. We have indicated this in the manuscript.

3. In the data collection section authors can clarify and add details on how interview tools were developed, translated and back translated, was it piloted before application?

**Response:** Interview guides were adopted from previous tools used for similar studies, translated into Afaan Oromo and Amharic but not back translated to English. The tools were also piloted as mentioned in the manuscript under Domain 2: Data collection section.

4. Authors should also describe the COVID related considerations that were applied during data collection in 2020?

**Response:** We have indicated this in the manuscript.

5. In the discussion, authors can comment about how the results/findings may be used to support implementation of the cRCT mentioned in the introduction.

**Response:** We have indicated this accordingly.

**Reviewer #2:**

1. Overall, this is an interesting manuscript that presents the results of a qualitative study evaluating perceptions of malnutrition among infants less than 6 month of age at two sites in Ethiopia. The results are interesting and may be of interest to those working in nutrition programs as well as primary care settings in LMIC settings.
   The abstract frames this work in a way that seems very narrow and limiting - understanding these challenges is important for many reasons - not just to inform research - I think the abstract sells the paper short and makes it feel far less relevant than it could. The idea that interventions only need to address misunderstandings and barriers to understanding is also problematic. I would strongly encourage the authors to take a broader view of this work and its implication for research, practice, policy.

**Response:** We have modified accordingly.

2. In the introduction, the authors suggest that supporting BF is the core of treatment. In may be worth noting that evidence from several studies (Mwagombe et al, etc) suggests that BF alone is not sufficient for recovery in severely malnourished infants.

**Response:** Yes, breastfeeding alone is not sufficient for recovery in severely malnourished infants. Our introductory point is reflecting that skilled breastfeeding support is a key component of case management of all breastfed malnourished infants, including those who are medically complicated and require inpatient care. However, as you state, it will not be sufficient for all. This is reflected in the MAMI Care Pathway approach that will be tested in the RCT trial, which includes IMCI-based clinical triage and feeding, clinical and maternal nutrition and health assessment and support. We have added a clarifying point to our introduction to make that clear.

3. In the last paragraph of the introduction - I am not sure I would structure this paper around the needs of the trial - that may be true, and worth mentioning, but as noted above the implications of this work are much broader.

**Response:** Thank you for the suggestion. We have modified accordingly.

4. Overall, more detail on how participants were selected (and a discussion of the inherent limitations to this approach) is needed.

**Response:** We have included the details under Domain 2, participants’ selection sub-section.

5. What is the difference between the face-to-face vs. in-depth interview?

**Response:** The phrase “face-to-face” is used to inform the readers that all key-informant and in-depth interviews were in-person and not remotely over the phone or through online. We have edited it now.

6. In the results section for Theme 1, there are a lot of behaviors being mentioned that are quite age dependent. A one month old has very different feeding, playing, eating behaviors than a 6 month old. Was there a sense that the participants recognized that these behaviors differed by age?

**Response:** Yes, it is true that infant behaviors vary by age. We did not explicitly examine this with the participants and so do not report on this.

7. On page 10, “Caregivers also consider that infants should sleep in a clean place where there are no insects.”. This seems more like a feature of the environment, not the child.

**Response:** That you for the comment, we have now deleted this statement.

8. Again, the lack of age appropriate behavior in the discussion is striking - was this evaluated? It seems very hard to interpret behaviors that differ by age.

**Response:** We agree that age-appropriate behaviour is an important consideration in this age group. Since we did not collect infant age data from participants, we cannot disaggregate and analyse the data by infant age.

9. Page 13, “there is a community belief that eating animal products and fruits will lead to excessive foetal growth and hence predispose to caesarean section delivery.” This is a well established phenomena - lots of data from Nepal about this - would be worth discussing more in detail.

**Response:** We have elaborated this accordingly in the discussion section.

10. Throughout the results sections (particularly in Theme 3), the authors present data as a synthesis of findings and not really results, please present objective findings in results and reserve the synthesis of findings for the discussion.

**Response:** We have modified the results and discussion sections accordingly.

11. On page 18, the authors note that there is confusing between nutritional status and feeding as well as confusion about BF. These are confusing to many people and it is not clear what this has to do with education. In addition, please move the discussion of why this may be to the discussion and present objective results only in results.

**Response:** We have modified as suggested.

12. For many of the HCW interviews it appears that there were some misconceptions. Important to note where the HCWs actually had erroneous impressions of what the guidelines say.

**Response:** We did not identify specific is conceptions or departures from guideline recommendations by HCWs. However, the interviews did identify that HCWs have gaps in skills in applying guidelines, including how to diagnose malnutrition among infants.

13. Page 22 – top of the Theme 5 section is repetitive with above section.

**Response:** Even if there seems to be overlap, theme 4 is about reasons for the occurrence of malnutrition among u6m infants whereas theme 5 presents about detection and treatment of malnutrition in infants u6m. Therefore, there is just continuity of information than overlaps or repetitions. However, there is a significant overlap between Theme 3 and Theme 5, which we have modified now.

14. Page 24 – please define abbreviations (HP – Health Post) when first using.

**Response:** We have defined accordingly.

15. Bottom of page 25 - This tendency to blame mothers for malnutrition is a major barrier and should be addressed and discussed. This leads to a breakdown of the critical alliance between HCWs and mothers and is a major issue.

Overall, it appears from these findings that education and knowledge are not the problem - mothers and HCWs knew what they were supposed to do, but did not do it. What other interventions are needed beyond KAP interventions?

**Response:** We have indicated this in discussion and conclusion sections. Interventions beyond KAP interventions include addressing the economic needs of lactating mothers and their families (since they are forced to introduce additional feeding early in order to get back to income earning activities), providing an extended period of maternity leave for lactating mothers, and providing comprehensive treatment, care and support for the lactating mothers (including mental health support to facilitate translation of the good knowledge and attitude into good practices).

**Reviewer #3:**

Thank you for the opportunity to review this manuscript which reports on a qualitative study “assess the perceptions and understanding of malnutrition in infants u6m and its management among carers, communities and healthcare workers in rural Ethiopia”. The study was conducted as formative research to inform a cluster randomized trial on management of malnutrition among young infants.

*Introduction*

1. “An estimated 8.5 million infants u6m worldwide are wasted (have low weight-for-length, a marker of severe malnutrition)” please clarify if you mean “An estimated 8.5 million infants u6m worldwide have weight-for-length Z scores <-3, a marker of severe malnutrition.” Ie is the 8.5 million among those with “moderate or severe wasting” or severe wasting only. Given the focus of the study, it would be best to present the data restricted to severe wasting.

**Response:** The 8.5 million is those with moderate and severe wasting combined, out of which 3.8million infants had severe wasting. We have indicated this in the respective section.

2. I believe the word conducive is intended rather than conductive in this phrase “requiring skilled professionals and a conductive environment”

**Response:** Yes, we meant “conducive”. We have corrected it accordingly.

3. In the list of practices and how prevalent they are on the top of page 5 “9% were given a bottle with a nipple” is listed in the midst of other practices related to TYPES of feeds – this items seems misplaced here.

**Response:** We have edited accordingly.

*Methods*

4. Domain 1 is well explained. My only question is related to the choice of the word ”authors” under personal characteristics. Did the entire research team co-author the manuscript or does the word choice need revision?

**Response:** Not all research team members are co-authors of the manuscript. We have replaced “Research team” by “co-authors”.

5. Domain 2 requires more context. For example either in the Methods or the Intro please describe health developmental army. HEWs, health managers, and program managers within the Ethiopian health system context. The readership will include persons outside of Ethiopia. For ex, are health managers the administrative heads of a district health management team? What is their positionality to program officers, etc.

**Response:** We have incorporated this in the relevant sections.

6. What is meant by carers? Are these caretakers of U6m? How were they and HDA members identified as interview candidates? I note the purposive sampling but we still need to understand how much bias is involved in the selection process. I’d like to know more about the eligibility criteria for all of the key informant types. Eg did you seek HCWs who manage sick and small infants? Were all parents approached because they had a child in a hospital?
   What is the difference between key-informant and in-depth interviews? Ie why the “or” in “key-informant interview or in-depth interview”

**Response:** Yes, by ‘carers’ we mean ‘caretakers’ of u6m infants that include mothers, fathers or the designated carer in their absence. We also interviewed HDAs who are community members who are closely engaged with carers of infants u6m. Those who have recent experience of caring for infants were purposively selected to minimize recall bias. Caregivers whose malnourished infants were enrolled in care in health centers or hospitals and who were willing to participate were include to capture diversity in experiences and situation. Available HDA leader or members were identified by the health extension workers and invited to participate in the study. HCWs who manage sick and small infants were sought and they participated in the study, including pediatricians at hospitals and family health unit coordinators at health centers who are also healthcare providers for this age-group.

Key-informant and in-depth interviews respectively refer to participants who can give information on behalf of others by virtue of their position or special knowledge (e.g elders, leaders, experts, etc ), and those who speak about themselves as members of a target group for study, in this instance caregivers in this study.

Domain 3 is well explained however, a statement as to whether a single coder per transcript was use or dual coding in which case how differences in coding between coders was resolved.

**Response:** A single coder per transcript initially coded which were shared with other team members for their reflections and feedback. The agreed upon versions were analyzed. We have described this in the relevant section.

Please submit a Consolidated criteria for reporting qualitative studies (COREQ) checklist as supplementary material. This will help the reader understand if this work was conducted according to standards for qualitative research.

**Response:** We have submitted the COREQ checklist.

*RESULTS*

Recommend merging the 1st 2 sentences: “We conducted a total of 31 interviews: 17 with mothers, fathers, and HDA leaders, and 14 with healthcare workers and managers (Table 1).” What about the program officers and HEWs that were mentioned in the methods? Are the mothers and fathers from the same household? How many health facilities are the HCWs from?

**Response:** We have modified accordingly. HEWs are already defined as part of HCWs. The mothers and fathers are not from the same household but from the same community. The HCWs were from 7 health facilities; health managers and program officers from 4 offices. All are incorporated in the manuscript.

~What % of HCWs in the Jimma and Deder are represented by the interviewees?
Are the descriptions of the 2 people who refused examples of refusals or the only 2? It’s a bit of an unusual way to report refusal rate.

**Response:** Given this is a qualitative study, statistical representation of study participants was not taken into consideration but variations in terms of access to health facilities were taken into account. Only two of the participants invited to take part on the interview (one father and one healthcare worker) refused to take part in the study.

Table 1 – In the methods section you note that the following types of health care workers were targeted for interviews: nurses, midwives, health officers, paediatricians, and HEWs. But Table 1 only includes the latter 2 which represent very different cadres. What about the nurses, midwives and HOs?

**Response:** We have modified accordingly.

Table 1 – were all of the fathers also HDA members? They are co-listed
Can you provide some basic demographic data about these interviewees? Eg ages, sex of HCWs

**Response:** Fathers were not HDA members. All HDA members are females. All HEWs are also female but other HCWs are mix of both sexes. We have modified the table 1 and also incorporated additional information.

Theme 1
It is difficult to understand how these very general interview responses on general health and development help us understand perceptions around management of severe malnutrition of U6m. The results are so general and sweeping but its hard to image how these will inform interventions.

**Response**: We have indicated how these behaviours will be helpful in the discussion section.

Theme 2
The data in the first section (p 13 and thru p 14 before the Reasons for early initiation of additional feedings section are hardly novel or surprising. The Reasons for early initiation of additional feedings section is more specific and adds to the literature and can help inform future work. The first bullet point re mother’s health and economic conditions deserves more elaboration.

**Response:** We have elaborated accordingly.

Theme 3 is an important section. But without more context on characteristics of the parents interviewed were are not able to fully appreciate these results. For ex, it is stated “Parents who actually had small or malnourished infants had a better understanding of related concepts because of their contact with health facilities during treatment.” But what % of parents interviewed had malnourished infants? See comments above re this issue.

**Response:** Characteristics of the study participants including parents are now elaborated in the methods section. Parents with or without malnourished infants were considered to capture variations in their knowledge and experiences given it is a qualitative study. Three out of 10 (30%) of the mothers interviewed had infants with malnutrition.

Treatment approaches as per the standard protocol – p 24 – is this the authors’ explanation of guidelines in Ethiopia or a report from the interviews? Is F75/F100 really recommended for management in <6 month olds and in health centers as opposed to in hospitals? Is there a reference for this guideline (if this is what it is)?

**Response:** What is mentioned in this section is the report from the interviews. It is important to note that in Ethiopia, some health centers have facility for admission and inpatient treatment; it is only in these health centers that therapeutic milks will be used. With regard to national guidelines, the Ethiopian Ministry of Health National guideline **(reference No. 13 in this manuscript)** recommends inpatient treatment (either at health centers having stabilization centers or hospitals having nutritional rehabilitation units) using F75 for those with edematous sever acute malnutrition and diluted F100 for those with non-edematous malnutrition.

Treatment related challenges
“HPs’ limited capacity to identify early-stage infant u6m malnutrition was reported as a key challenge.” – reported by which type of interviewees?

**Response:** This is reported by the HCWs at the health centers.

2nd paragraph – again, I’m not sure if this information about norms is supposed to be background provided by the authors or if they are reporting data from their interviews.

**Response:** We are reporting this from the interview findings.

*Discussion*

Through the bottom of page 29 most of the text is a rehash of the results with little suggestion of how these data are helpful in informing their future RCT, for example. Top of page 30 is a description of other literature. My point is that the discussion does not synthesize the results in a way that helps the reader understand how these results can be used, how they can inform next steps and additional work. The aim of the study was noted to inform a cluster randomized trial on management of malnutrition among young infants but it's not clear how - this requires some explanation.

**Response:** We have modified the content.

Bottom of p 29 – “carers often prefer health centres, hospitals and other higher-level facilities due to a perception that better treatments are available there” – compared to what?? Community based care?

**Response:** We have revised it.

A thorough proofreading of the manuscript is necessary to catch all grammatical errors, some examples: in the Ethics section: “We obtained written consent from all study participants with language of their interest”. Another example from the Introduction where there is a tense mismatch within the sentence: “Whilst exclusive breastfeeding was practiced by 59% of mothers overall, declining sharply with age:”. Another example from the results section: “A common community belief says that infants cry for food looking when others feed”

**Response:** We have edited accordingly.
